# Cohort profile: follow-up of a household cohort throughout five epidemic waves of SARS-CoV-2 in Rio de Janeiro, Brazil

**DOI:** 10.1590/0102-311XEN152023

**Published:** 2024-08-26

**Authors:** Michele Fernanda Borges da Silva, Lusiele Guaraldo, Heloisa Ferreira Pinto Santos, Leonardo Soares Bastos, Anielle de Pina-Costa, Stephanie Lema Suarez Penetra, Isabella Campos Vargas de Moraes, Guilherme Amaral Calvet, Trevon Louis Fuller, Paola Cristina Resende Silva, Marilda Agudo Mendonça Teixeira de Siqueira, Patricia Brasil

**Affiliations:** 1 Instituto Nacional de Infectologia Evandro Chagas, Fundação Oswaldo Cruz, Rio de Janeiro, Brasil.; 2 Programa de Computação Científica, Fundação Oswaldo Cruz, Rio de Janeiro, Brasil.; 3 University of California, Los Angeles, U.S.A.; 4 Instituto Oswaldo Cruz, Fundação Oswaldo Cruz, Rio de Janeiro, Brasil.

**Keywords:** Coronavirus Infections, SARS-CoV-2, Cohort Studies, Natural History of Diseases, Infecções por Coronavírus, SARS-CoV-2, Estudos de Coortes, História Natural das Doenças, Infecciones por Coronavírus, SARS-CoV-2, Estudios de Cohortes, Historia Natural de las Enfermedades

## Abstract

Since May 2020, we have been conducting a comprehensive study to understand the natural history of SARS-CoV-2 infection in Rio de Janeiro, Brazil. Our focus has been on following families, systematically collecting respiratory tract swabs and blood samples, monitoring symptoms, and gathering data on vaccine status. This paper aims to describe the household cohort across five epidemic waves of SARS-CoV-2, providing an overview of the collected data and a description of the epidemiological, clinical, and immunological characteristics and incidence of SARS-CoV-2 infection. Our cohort includes 691 participants from 189 households. During the five epidemic waves, we detected 606 infections. The incidence density of SARS-CoV-2 infection ranged from 4 (Delta) to 56 (B.1.1.33) per 1,000 person-week, with a peak in wave B.1.1.33 in all age groups. The seroprevalence of SARS-CoV-2 antibodies (IgG anti spike protein) varied from 37%, in the pre-VoC period, to 99%, in the Omicron period, progressively increasing after each wave in a similar manner regardless of age. As we have monitored the cohort continuously since the beginning of the pandemic, we were able to collect data across different scenarios according to the predominant lineage in circulation. Via active monitoring of families, we were able to carry out an epidemiological surveillance on SARS-CoV-2, including its variants, persistence of symptoms, and changes in immunity over time in the population, contributing to knowledge of the natural history of SARS-CoV-2 infection.

## Resumo

Desde maio de 2020, temos conduzido um estudo abrangente para entender a história natural da infecção por SARS-CoV-2 no Rio de Janeiro, Brasil. Nosso foco tem sido acompanhar as famílias das quais coletamos sistematicamente amostras de sangue e do trato respiratório, monitoramos os sintomas e reunimos dados sobre o status de vacinação. Este artigo tem como objetivo descrever a coorte de domicílios ao longo de cinco ondas epidêmicas de SARS-CoV-2, fornecendo uma visão geral dos dados coletados e uma descrição das características epidemiológicas, clínicas e imunológicas e da incidência da infecção por SARS-CoV-2. Nossa coorte inclui 691 participantes de 189 domicílios. Ao longo das cinco ondas epidêmicas, detectamos 606 infecções. A densidade de incidência da infecção por SARS-CoV-2 variou de 4 (Delta) a 56 (B.1.1.33) a cada 1.000 pessoas por semana e foi mais alta na onda B.1.1.33 em todas as faixas etárias. A soroprevalência de anticorpos contra o SARS-CoV-2 (proteína IgG anti-spike) variou de 37% no período pré-VoC a 99% no período Omicron e aumentou onda após onda de maneira semelhante, independentemente da idade dos participantes. Como monitoramos a coorte continuamente desde o início da pandemia, pudemos coletar dados em diferentes cenários, de acordo com a cepa predominante em circulação. Por meio do monitoramento ativo das famílias, conseguimos conduzir uma vigilância epidemiológica do SARS-CoV-2, de suas variantes, da persistência dos sintomas e das mudanças na imunidade da população ao longo do tempo, contribuindo para o conhecimento da história natural da infecção pelo SARS-CoV-2.

## Resumen

Desde mayo de 2020 se realiza un estudio exhaustivo con el fin de estimar el curso natural de la infección por SARS-CoV-2 en Río de Janeiro, Brasil. Se aplica un seguimiento a las familias en el cual se recolectan sistemáticamente muestras de sangre y de las vías respiratorias, se controlan los síntomas y se recogen datos sobre el estado de vacunación. Este artículo tiene como objetivo describir la cohorte de hogares durante cinco olas epidémicas de SARS-CoV-2, y proporcionar una visión general de los datos recopilados y una descripción de las características epidemiológicas, clínicas e inmunológicas, y de la incidencia de la infección por SARS-CoV-2. La cohorte incluyó a 691 participantes de 189 hogares. A lo largo de las cinco olas epidémicas, se detectaron 606 infecciones. La densidad de incidencia de la infección por SARS-CoV-2 varió de 4 (Delta) a 56 (B.1.1.33) por cada 1.000 personas por semana, y fue más alta en la ola B.1.1.33 en todos los grupos de edad. La seroprevalencia de anticuerpos contra el SARS-CoV-2 (proteína IgG antipico) varió del 37% en el período anti-VOC al 99% en el período Ómicron y aumentó ola tras ola de manera similar, independientemente de la edad de los participantes. El monitoreo continuo de la cohorte desde el comienzo de la pandemia permitió recopilar datos en diferentes escenarios según la cepa predominante en circulación. A partir del monitoreo activo de las familias, se realizó una vigilancia epidemiológica del SARS-CoV-2, sus variantes, la persistencia de los síntomas y los cambios en la inmunidad de la población a lo largo del tiempo, contribuyendo al conocimiento del curso natural de la infección por SARS-CoV-2.

## Background

Since February 2020, more than 37 million people have been infected with SARS-CoV-2 in Brazil. From February 2020 to March 2021, more than 3,000 deaths were recorded due to COVID-19 in Brazil every day. The total number of deaths attributed to COVID-19 in Brazil is more than 700,000. In the state of Rio de Janeiro, 2.8 million SARS-CoV-2 cases and 77,000 deaths have been reported as of July 2023. Rio de Janeiro’s COVID-19 fatality rate of 447 per 100,000 inhabitants is the highest among Brazilian states. While extraordinarily high, these estimates may actually be conservative due to underreporting and the scarcity of diagnostic tests ^1^.

A variety of SARS-CoV-2 lineages have been identified in Rio de Janeiro. The first to become predominant was B.1.1.33 in March 2020 followed by the Zeta (P.2) variant in October 2020, Gamma (P.1) in January 2021, Delta (B.1.617.2/AY.*) in June 2021, and Omicron (BA.*) in December 2021 ^2^. Social distancing measures were first adopted in March 2020, including quarantine and school closures. In June 2020, mask-wearing became mandatory. However, the population displacement index showed that only 47.7% of the state’s population adhered to the recommendations. In July 2020, restaurants, gyms, and hotels were able to reopen with restrictions, and only six months later, in January 2021, vaccination began ^3^ ^,^ ^4^.

To better elucidate the natural history of SARS-CoV-2 infection, since May 2020 we have been following families, systematically collecting respiratory tract swabs and blood samples and monitoring symptoms and vaccine status. This paper aims to comprehensively describe the household cohort across the five SARS-CoV-2 epidemic waves, providing an overview of the data collected and a description of the cohort participants’ epidemiological, clinical, and immunological characteristics and the incidence of SARS-CoV-2 infection.

## Material and methods

### Study population and participant recruitment

The cohort participants are residents of the Rio de Janeiro metropolitan area, Brazil, a political-spatial section formed by 22 municipalities. It covers 12% of the state’s area and holds a population of approximately 12 million inhabitants ^5^.

The cases of COVID-19 (index cases) were selected at the outpatient clinic of the Evandro Chagas National Institute of Infectious Disease, Oswaldo Cruz Foundation (FIOCRUZ), Rio de Janeiro, a public clinic that is an infectious diseases referral center, as well as a clinical research and teaching center. During the pandemic, treatment was provided to all patients who arrived at the clinic seeking care. All individuals who agreed to participate in the study signed an informed consent form. Those aged from six to 17 provided written assent to participate in the study.

### Cohort description and definitions

This prospective study is an open cohort of individuals with laboratory-confirmed SARS-CoV-2 infection and their household contacts who have been followed since May 2020.

Inclusion criteria were individuals infected with SARS-CoV-2 confirmed by reverse transcription-polymerase chain reaction (RT-PCR) test within 10 days of the onset of symptoms and their household contacts.

Participants were included regardless of being symptomatic or asymptomatic. The study was designed following the World Health Organization (WHO) protocols to provide rapid and early information on the clinical, epidemiological, and virological characteristics of SARS-CoV-2 infections in communities ^6^.

The index cases were a convenience sample of patients at the outpatient clinic. Sequential sampling was used to recruit participants based on their arrival at the clinic, provided they met the eligibility criteria. An index or primary case was defined as the household member who first experienced symptoms and had a positive RT-PCR test for SARS-CoV-2. When two or more family members had the same onset date of COVID-19-like symptoms, they were defined as co-primary cases.

Later cases were classified as secondary cases, namely, household members with symptoms or RT-PCR positive to SARS-CoV-2 tests within 2-14 days of an index case. Household contacts were any individual residing in the same household, including domestic workers (such as maids, nannies, and caregivers) and people living on the same plot of land or who shared common areas.

Asymptomatic infections were defined as the absence of symptoms in individuals with positive results for SARS-CoV-2 based on an RT-PCR test until the test was negative. Pre-symptomatic infections were defined as the presence of a positive test before onset of symptoms ^7^ ^,^ ^8^.

Reinfection cases were defined by two COVID-19 episodes in the same individual by distinct SARS-CoV-2 lineages detected or inferred by laboratory assays (whole genome sequencing [WGS] or RT-PCR inference assay). In cases of suspected reinfection and viral load insufficient for WGS, the case was classified as reinfection if the new infection occurred over 90 days after the previous infection, in addition to a SARS-CoV-2 negative RT-PCR test between the two episodes ^9^ ^,^ ^10^.

Incidence density was defined as the number of new SARS-CoV-2 cases per 1,000 person-week of exposure. For each period, the disease-free observation period for each participant was determined by the duration of observation during that specific period, plus two additional weeks. The two extra weeks were included to account for the maximum interval for SARS-CoV-2 transmission and the detection of infection by the study team. If follow-up spanned multiple periods, the at-risk time was truncated at the beginning and end of each period.

To estimate seroprevalence, those with positive confirmed serology were considered as numerator, defined as having at least one positive serology test (anti-nucleocapsid Ab or anti-spike Ab), with the total number of participants being considered in the denominator. Incidence density and seroprevalence were estimate for each epidemic wave. Epidemic waves were defined based on the predominant circulating SARS-CoV-2 lineage according to genomic surveillance ^2^.

For georeferencing the households, Google Maps (https://www.google.com.br/maps/preview) was used to obtain latitude and longitude information based on the address provided by the participant. Then, this information was entered into the ArcGIS software (http://www.esri.com/software/arcgis/index.html) to create the map. The seroprevalence by region in each epidemic wave was compared using analysis of variance (ANOVA) (Supplementary Material, Figure S1: https://cadernos.ensp.fiocruz.br/static//arquivo/suppl-csp-1520-23_1888.pdf).

### Data collection and follow-up

Baseline and follow-up data were collected and managed using the REDCap software (https://redcapbrasil.com.br/) ^11^ ^,^ ^12^. In total, 602 variables were collected, accounting for sociodemographic, behavioral, clinical, and immunization data, as well as laboratory data such as the results of RT-PCR tests, viral genome sequencing, and serology assays. Information on hospitalization and the use of medication was also obtained whenever possible. After being entered into the REDCap, queries of the database were performed with MySQL Workbench software version 8.0 (https://dev.mysql.com/downloads/workbench/). Data quality control was conducted weekly. The data were shared securely via the ownCloud platform, which holds a RNP ICPEdu OV SSL CA 2019 security certificate. Supplementary Material, Figure S2 (https://cadernos.ensp.fiocruz.br/static//arquivo/suppl-csp-1520-23_1888.pdf) shows the structure of the database.

Follow-up of signs and symptoms, hospitalizations, vaccination, and health behavior were conducted via systematic telemonitoring by phone calls according to the study schedule. Home visits were performed to collect biological material (blood, nasopharyngeal swabs, and saliva). Box 1 summarizes the data collected at each visit.

**Box 1:** Schedule of data collection in the cohort.

DATA	DAYS OF ONSET SYMPTOMS *													EXTRA **
	7	14	21	42	90	120	180	270	360	450	540	630	720	
Telemonitoring														
Sociodemographic														
Age, sex, ethnicity, schooling level, profession, address.														
Household information														
Number of household contacts														
Relationship of contact between index case														
House information (size, room number)														
Contact with COVID-19 case														
Sleeping in the same room														
Health behavior														
COVID-19 transmission-prevention behavior (including physical distancing, hygiene, and use of physical barriers)														
COVID-19 testing and self-isolation														
Health information														
Comorbidities														
Current medication														
Health-related behavior (tobacco use)														
Symptoms														
Medical assistance														
Hospitalization														
Vaccination (number of doses, dates, types)														
Home visits														
Laboratory tests														
Serology (SARS-CoV-2 IgG)														
RT-PCR SARS-CoV-2 (saliva)														
RT-PCR SARS-CoV-2 (nasopharynx)														

* With an acceptable delay of seven days (until 42 days) and 14 days, approximately (after 42 days);

** For COVID-19 symptoms, contact with a SARS-CoV-2 positive individual, or increase in the average number of cases in the state population.

Sample collection and clinical follow-up was performed systematically based on the onset of symptoms of the index case or the date of immunization. These follow-ups were conducted every 7-14 days for the first 42 days after diagnosis of infection by RT-PCR. Subsequent follow-up was conducted every 90 days for 24 months.

Clinical appointments and additional sample collections were performed whenever the participant reported persistence of symptoms (> 7 days), new onset of symptoms, or contact with a suspected/confirmed COVID-19 case.

### Laboratory data

Upper respiratory tract (nasopharynx and oropharynx) swabs were collected and stored in viral transport medium. Saliva samples were collected under nurses’ supervision, by asking the participant to accumulate saliva (at least 1-2mL) in the mouth for one minute and then drooling into a sterile container without coughing or clearing their throats ^13^.

All samples were screened for SARS-CoV-2 by real-time RT-PCR to amplify the E gene and the RdRp region of the Orf1ab gene, following the Charité protocol (TC < 38) (Charité/Berlin, Germany). A cycle threshold (CT) value of less than 40 was considered positive. SARS-CoV-2 genomes were obtained from the Illumina amplicon sequencing using the QIAGEN CLC Genomics Workbench software version 20.0.4 (https://digitalinsights.qiagen.com/products-overview/discovery-insights-portfolio/analysis-and-visualization/qiagen-clc-genomics-workbench/). Phylogenetic relationships among SARS-COV-2 samples were determined using the Pango Lineages tool.

To characterize the viral lineages, whole genome sequencing were generated either by an in-house protocol with the 2-kilobase 17 amplicon tiling strategy ^14^ or the Illumina COVIDSeq Test protocol (Illumina) containing the primer set ARTIC V3 or V4 with adaptations made by the Fiocruz COVID-19 Genomic Surveillance Network using the Illumina MiSeq or NextSeq 2000 sequencer ^15^. FASTQs were retrieved from the BaseSpace Illumina, and the genomes were assembled using the ViralFlow pipeline (https://github.com/dezordi/ViralFlow) ^16^. The genome mutation profile was analyzed with the CovSurver (https://mendel.bii.a-star.edu.sg/METHODS/corona/beta/) and NextClade (https://clades.nextstrain). Additionally, the SARS-CoV-2 lineages were classified using the PangoLineages tool ^17^. Variants of concern (VoCs) were identified using a real-time RT-PCR inference assay (Institute of Technology on Immunobiologicals [Bio-Manguinhos]/FIOCRUZ). This assay detects the virus using the N and RP targets and additionally detects the presence or absence of the deletions S106del, G107del, and F108del in the ORF1a gene (nsp6) and the spike gene target failure (SGTF) at the positions H69del and V70del. Combining the presence or absence of these mutations with the epidemiological scenario allowed us to identify the SARS-CoV-2 VoC.

Blood samples were collected for humoral immunity tests. Serum was screened for IgG antibodies via Microparticle Chemiluminescent Immunoassay (CMIA), anti-nucleocapsid Ab, and anti-spike Ab (Abbott Laboratories, https://www.abbott.com/). All assays were performed following the manufacturer’s instructions.

### Ethics approval

The study was approved by the Brazilian National Research Ethics Committee (CONEP, n. 30639420.0.0000.5262).

## Results

**Figure 1:**
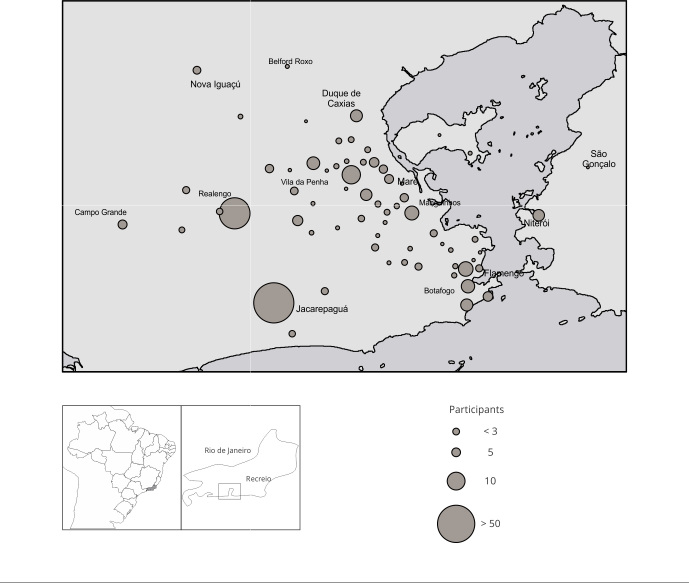
Distribution of study participants by neighborhood in Rio de Janeiro, Brasil, and surrounding municipalities.

As of April 2023, 708 individuals met the eligibility criteria and were invited to participate in the study. A total of 691 individuals from 189 households agreed to participate in the study, with a median of three individuals per household. In total, 11 contacts refused to participate either stating discomfort during swab collection or failing to provide a reason for their refusal. Figure 1 shows the number of participants by neighborhood in Rio de Janeiro and surrounding municipalities. At baseline, 189 participants were index cases, and 18 were co-primary cases.

During the monitoring period, the same individual could be an index case more than once, and 288 participants were classified as primary cases at least once. Of the 502 household contacts, 49% were infected with SARS-CoV-2.

**Figure 2:**
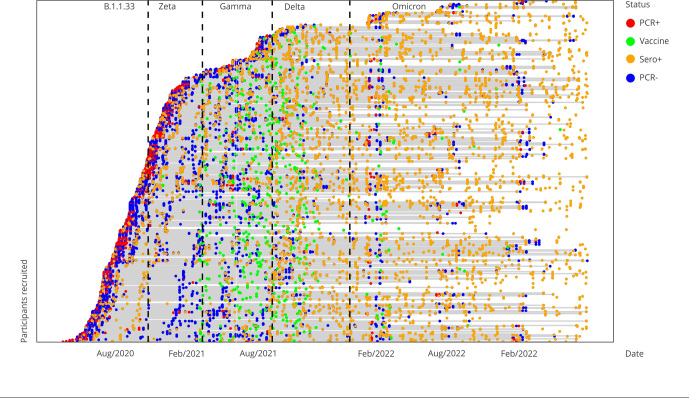
Virological and immunological results for each cohort participant.

Most families (53.7%) were enrolled during the first epidemic wave. Enrollment during the Zeta, Gamma, Delta, and Omicron waves was considerably lower (at 24.6%, 11.4%, 3.2%, and 7.1%, respectively). The proportion of participants retained in the study (those who collected at least one biological sample during the period) ranged from 54% to 68.2% in the different epidemic waves. The median duration of follow-up was 736, 740, 630, 300, and 149 days, respectively. Figure 2 shows the duration of follow-up, the results of real-time RT-PCR and serological tests, and data on the immunization of participants.

A total of 10,536 telemonitoring visits, 6,622 RT-PCR tests, and 7,925 serology tests were performed during the study period. From May 2020 to April 2023, 606 infections were detected, of which 22.8% were reinfections. There were 50 participants (7.2%) who, upon cohort entry, were N antibody negative, indicating that they had neither been infected with SARS-CoV-2 nor vaccinated. Of the total infections followed in the cohort 95 (15.7%) were asymptomatic and 34 (5.6%) were pre-symptomatic and developed symptoms within a median of 4 (range: 1-13) days after testing positive.

The median age of the participants was 37 years (interquartile range − IQR: 21-53). Most participants were adults (80.2%), women (54.7%), self-reported as white (56%), with complete secondary education (83%), and 13% were healthcare professionals. Table 1 shows the sociodemographic profile of the study population.

**Table 1:** Sociodemographic and clinical characteristics of the study participants.

Sociodemographic and clinical characteristic	N = 691 (%)
Age [median (IQR)]	37 (21-53)
0-4	37 (5.4)
5-11	58 (8.4)
12-17	42 (6.1)
18-39	243 (35.2)
40-59	210 (30.4)
60+	101 (14.6)
Sex	
Female	378 (54.7)
Male	313 (45.3)
Profession	
Healthcare worker	90 (13.0)
Non-healthcare worker	601 (87.0)
Skin color/Ethnicity	
White	386 (56.0)
Non-white	302 (43.7)
Unknown/Refused to answer	3 (0.4)
Comorbidities	422 (61.1)
Systemic arterial hypertension	154 (36.5)
Rhinitis	89 (21.1)
Overweight/Obesity	76 (18.0)
Metabolic disease	57 (13.5)
Chronic lung disease	55 (13.0)
Neuropsychiatric disease	43 (10.2)
Thyroid disease	37 (8.8)
Cardiovascular disease	26 (6.2)
Respiratory disease	21 (5.0)
Immunosuppressive therapy	20 (4.7)
Other chronic diseases *	35 (8.3)
Other diseases or health conditions **	21 (5.0)
Current smoker or ex-smoker	82 (19.4)
Hospitalization	22 (3.2)
Death	3 (0.4)
Vaccine status ***	
Fully vaccinated	624 (90.3)
1st booster	472 (68.3)
2nd booster	306 (44.3)
3rd booster	54 (7.8)
Partially vaccinated	28 (4.1)
Unvaccinated	39 (5.6)
Schooling level ^#^	
Never attended school or incomplete middle school	32 (5.8)
Complete middle school or incomplete high school	48 (8.7)
Complete secondary education or incomplete higher education	252 (45.5)
Complete higher education	207 (37.4)
Unknown/Refused to answer	17 (3.1)

IQR: interquartile range.

* Rheumatological disease (11), hematological disease (7), kidney disease (6), liver disease (4), osteoporosis (4), Chagas disease (2), and genetic disease (1);

** Gastrointestinal disorder (11), labyrinthitis (1), stroke sequelae (4);

*** Among participants eligible for vaccination;

^#^ Adult participants only.

Among 691 participants, 61.1% had at least one underlying medical condition. The most common comorbidities were systemic arterial hypertension, followed by rhinitis, being overweight or obese, and metabolic disease (diabetes mellitus and dyslipidemia). A total of 19.4% of the participants reported prior or ongoing tobacco use.

In total, 90% of the participants were considered fully vaccinated, either because they had received two doses of the whole inactivated Sinovac Biotech/Butantan Institute vaccine, the Oxford University/Bio-Manguinhos ChAdOx1-S/nCoV-19 adenovirus vector vaccine, or the Pfizer/BioNTech BNT162b2 mRNA vaccine or one dose of the Jansen Ad26.COV2.S vaccine. Most vaccination occurred in the first half of 2021. In total, 68.3% of the participants received a first booster (Ad26.COV2.S or BNT162b2), 44.3% a second booster (Ad26.COV2.S or BNT162b2), and 7.8% a third booster with bivalent (ancestral and Omicron BA.4/BA.5) mRNA vaccine (BNT162b2). The unvaccinated individuals (5.6%) reported vaccine hesitancy or mistrust.

Table 2 summarizes the incidence density of SARS-CoV-2 and seroprevalence of all participants. Moreover, 49.3% of the included participants were infected by SARS-CoV-2 during the first wave, 28.8% during the Zeta wave, 17.4% during the Gamma wave, 7.3% during the Delta wave, and 48.1% during the Omicron wave. The highest incidence occurred in wave B.1.1.33 for all age groups, with 29.4 per 1,000 person-weeks for individuals aged 0 to 17 years and 64.9 per 1,000 person-weeks for participants aged 60 and older. During the five epidemic waves, adults aged 18 to 59 were the most affected.

**Table 2:** Incidence density and seroprevalence of SARS-CoV-2 in the cohort across the five epidemic waves according to age group.

Epidemic wave	All participants			Participants by age group (years)								
				0-17			18-59			> 60		
	n	RT-PCR+	Incidence density *	n	RT-PCR+	Incidence density *	n	RT-PCR+	Incidence density *	n	RT-PCR+	Incidence density *
B.1.1.33	373	184	56	79	22	29.4	244	132	61.8	50	30	64.9
Zeta	454	131	22	91	16	13.8	294	94	24.1	69	21	23
Gamma	471	82	10	86	14	9.1	309	56	9.98	76	12	8.46
Delta	461	34	4	76	8	5.78	304	22	3.81	81	4	2.55
Omicron	395	190	18	74	28	13.5	259	139	20.6	62	23	12.7
	n	Sero+ **	%	n	Sero+ **	%	n	Sero+ **	%	n	Sero+ **	%
B.1.1.33	309	115	37	54	10	18.5	210	88	41.9	45	17	37.8
Zeta	331	149	45	62	14	22.6	218	108	49.5	51	27	52.9
Gamma	456	216	47	71	15	21.1	308	156	50.6	77	45	58.4
Delta	460	434	94	67	53	79.1	313	304	97.1	80	77	96.3
Omicron	464	460	99	67	65	97	319	318	99.7	78	77	98.7

n: number of participants assessed in each wave.

* per 1,000 person/week;

** At least one seropositive test during the period.

The seroprevalence ranged from 37% (pre-VoC period) to 99% (Omicron wave). The highest seroprevalence was observed in adults and older adults, progressively increasing after each wave in a similar manner. The results indicated no significant difference between regions and neighborhoods during any given wave (p > 0.05). However, seroprevalence progressively increased after each wave (Supplementary Material, Figure S1: https://cadernos.ensp.fiocruz.br/static//arquivo/suppl-csp-1520-23_1888.pdf).

## Discussion

The incidence density of SARS-CoV-2 infection in this prospective household cohort in Rio de Janeiro ranged from 4 (Delta) to 56 (B.1.1.33) per 1,000 person-weeks across the five epidemic waves, with the highest incidence among working age adults. The incidence of SARS-CoV-2 infection in our first wave greatly surpasses the rates reported in cohorts in the United States, which ranged from 5.1 (95%CI: 3.3-7.8) to 12.9 (95%CI: 8.6-17.4) ^18^ ^,^ ^19^. The difference among the incidence rates might be explained by different study designs, different definitions of SARS-CoV-2 infection (symptomatic and asymptomatic infections), and the sociodemographic characteristics of the populations studied.

Among the factors that may have contributed to the higher incidence rate are the inclusion of COVID-19 cases confirmed by RT-PCR and active searching for SARS-CoV-2 infections among household contacts. Moreover, the incidence rate may have been higher in this study because we followed the participants for more than two years, during which they were contacted regularly to enquire about COVID-19 symptoms and their household contacts were also diagnosed. A recent prospective cohort study conducted in a low-income community in Rio de Janeiro reported a similar incidence rate of SARS-CoV-2 infection of 52.5 per 1,000 person-weeks during the first wave of the pandemic ^20^. The two studies may have reported similar incidence rates due to being conducted in communities with similar socioeconomic conditions, used the same definition of infection, and employed similar designs for monitoring participants.

Although the highest incidence occurred in the first wave, the number and proportion of infections was higher during the Omicron wave. Factors that might explain this include the extended duration of the Omicron wave compared to previous ones, as well as its increased transmissibility compared to prior VoCs. Moreover, during the Omicron wave, the participants may have also experienced a decline in humoral immunity, increasing their susceptibility to infection.

The seroprevalence of SARS-CoV-2 antibodies (IgG anti spike protein) varied from 37% during the period of B.1.1.33 to 99% during the period of Omicron predominance, likely reflecting prior exposures to the virus followed by the rollout of SARS-CoV-2 vaccination, respectively. The prevalence of SARS-CoV-2 antibodies among our study participants was similar to that of serosurvey conducted in Brazil at the national scale ^21^. However, in the United States, the seroprevalence of SARS-CoV-2 in the first year of the pandemic and Omicron wave was lower, ranging from 3.5% to 64.3% estimated in blood donors ^22^ ^,^ ^23^ and 8% and 58.2% of the population of all 50 states ^24^ ^,^ ^25^, respectively.

The proportion of individuals infected decreased from 49.3% in the first wave to 7.3% in the Delta wave. rising again to 48.1% in the Omicron wave. As noted above, this may be partly attributable to Omicron capacity to escape from the immune response due to antigenic divergence and waning levels of neutralizing antibodies after infection and/or immunization ^26^.

At 90.3%, the rate of vaccination in our cohort was higher than the national average in Brazil ^27^. Nevertheless, vaccination rates in Brazil are relatively high, with refusal rates from 10.5% ^17^ to 17.5% ^18^. Individuals refusing vaccination typically stated mistrusting vaccines in general or COVID-19 vaccines in particular. The above-average vaccination rate in our cohort may be attributable to the participants’ willingness to accept scientific information.

The frequency of comorbidities (61%) in this cohort − the most common of which were hypertension, obesity, and diabetes − is similar to previous cohort studies and health surveys conducted in Brazil ^28^ ^,^ ^29^. Nevertheless, 3.2% of the participants were hospitalized and 0.4% died. However, most of our COVID-19 cases were mild (96%). We estimated that 15.7% of SARS-CoV-2 infections were asymptomatic and 5.6% pre-symptomatic, which is consistent with findings from a systematic review encompassing studies involving 20,152 cases (13.34% asymptomatic and 7.64% pre-symptomatic individuals) ^30^. Since the beginning of the pandemic, the rate of asymptomatic SARS-CoV-2 infection has been widely discussed due to its potential impact on transmission. However, rates of asymptomatic cases vary significantly depending on the population and design of the studies. A limitation of cross-sectional studies is that they can only evaluate the symptom profile of individuals at the time of testing, hindering differentiation between truly asymptomatic cases and those that are pre-symptomatic ^7^.

The limitations of our study were: firstly, due to the manner in which the cohort participants were selected, we cannot guarantee that they are representative of the metropolitan area of Rio de Janeiro; secondly, the declining retention of participants in the course of the study, which is characteristic of cohort studies. In this study, retention decreased mainly after relaxation of social distancing measures and the rollout of vaccination. However, we attempted to mitigate the loss of participants by maintaining frequent phone calls and home visits.

Strengths of this study include its prospective study design and longitudinal follow-up since the beginning of the pandemic. Our study design enabled data collection across different scenarios according to the predominant lineage in circulation. By monitoring families actively, we were able to conduct epidemiological surveillance of SARS-CoV-2, including its variants, persistence of symptoms, and changes in immunity over time in the population, contributing to knowledge of the natural history of the disease.

After three years since the declaration of a public health emergency by the WHO ^31^, SARS-CoV-2 virus infection continues to pose new challenges to the healthcare system that require constant monitoring and evaluation. These challenges include the risk of long COVID. We believe that future research could capitalize on the data collected in this study to gain insights into long COVID. We followed the study participants prospectively, monitored the persistence of their signs and symptoms, and collected biological samples systematically. In future work, it may be possible to use these data to identify clinical, epidemiological, serological, and immunological markers that are predictive of long COVID and estimate its incidence.
