# Supplementary material for: Cohort profile: follow-up of a household cohort throughout five epidemic waves of SARS-CoV-2 in Rio de Janeiro, Brazil
Source: Cad Saude Publica. 2024 Aug 26;40(7):e00152023. doi: 10.1590/0102-311XEN152023 (PMC11349275; doi:10.1590/0102-311XEN152023)
Supplement: Supplementary file 1 [file 1678-4464-csp-40-07-EN152023-s.pdf]

Supplementary Material

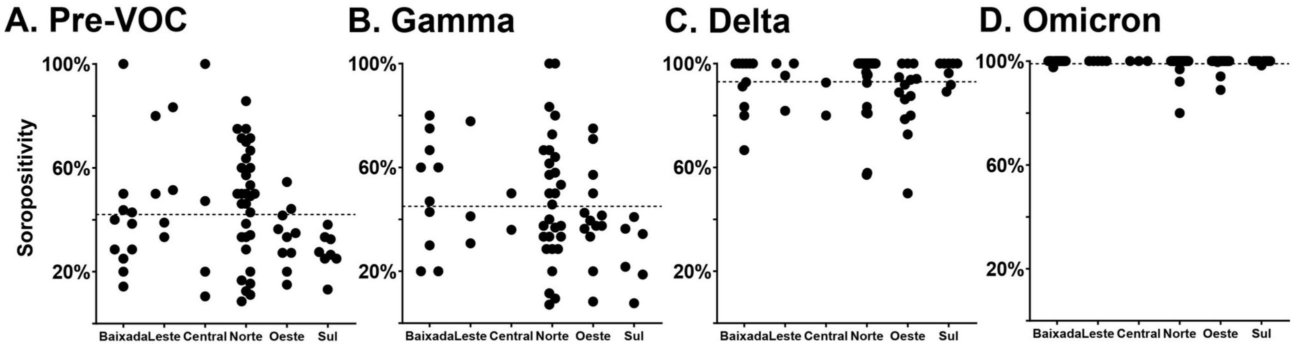

Figure S1 Seroprevalence by region in Rio de Janeiro, Brazil, in each epidemic wave.

Each panel of the figure represents an epidemic wave, at each point a neighborhood. The neighborhoods are organized by geographic region of Rio de Janeiro. In each wave, the horizontal line shows the median seroprevalence.

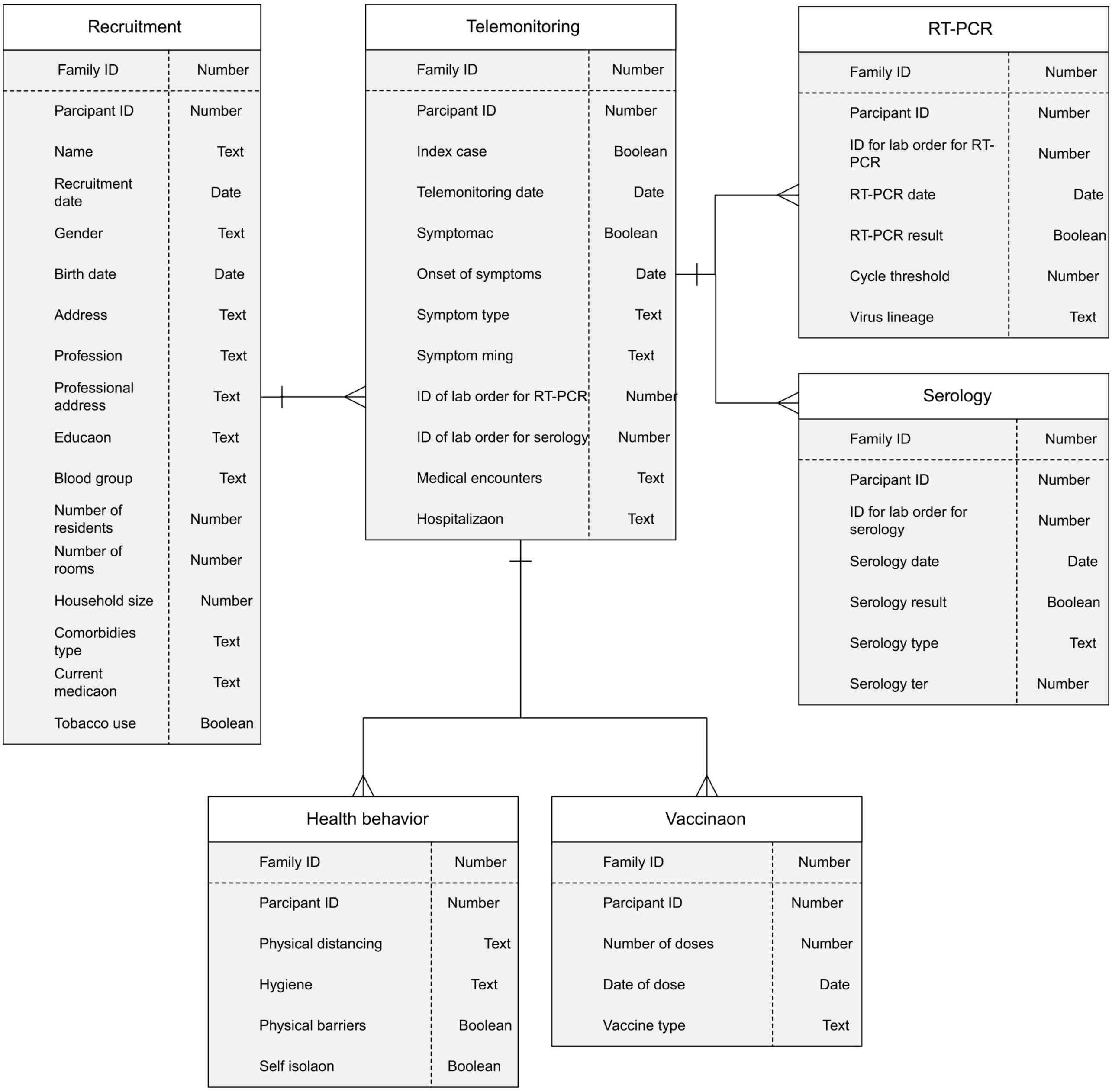

Figure S2 Participants' database structure.
